# Analgesic Effect of Melittin on Oxaliplatin-Induced Peripheral Neuropathy in Rats

**DOI:** 10.3390/toxins11070396

**Published:** 2019-07-08

**Authors:** Seunghwan Choi, Hyeon Kyeong Chae, Ho Heo, Dae-Hyun Hahm, Woojin Kim, Sun Kwang Kim

**Affiliations:** 1 Department of East-West Medicine, Graduate School, Kyung Hee University, Seoul 02447, Korea; 2 Department of Science in Korean Medicine, Graduate School, Kyung Hee University, Seoul 02447, Korea; 3 Anapn Korean Traditional Medical Clinic, 11, Seongnae-ro, Gangdong-gu, Seoul 05392, Korea; 4 Department of Physiology, School of Medicine, Kyung Hee University, Seoul 02447, Korea; 5 Department of Physiology, College of Korean Medicine, Kyung Hee University, Seoul 02447, Korea

**Keywords:** chemotherapy, cold allodynia, mechanical allodynia, melittin, neuropathic pain, oxaliplatin

## Abstract

Oxaliplatin is a chemotherapeutic agent used for metastatic colon and other advanced cancers. Most common side effect of oxaliplatin is peripheral neuropathy, manifested in mechanical and cold allodynia. Although the analgesic effect of bee venom has been proven to be effective against oxaliplatin-induced peripheral neuropathy, the effect of its major component; melittin has not been studied yet. Thus, in this study, we investigated whether melittin has an analgesic effect on oxaliplatin-induced allodynia. Intraperitoneal single injection of oxaliplatin (6 mg/kg) induced mechanical and cold allodynia, resulting in increased withdrawal behavior in response to von Frey filaments and acetone drop on hind paw. Subcutaneous melittin injection on acupoint ST36 (0.5 mg/kg) alleviated oxaliplatin-induced mechanical and cold allodynia. In electrophysiological study, using spinal in vivo extracellular recording, it was shown that oxaliplatin-induced hyperexcitation of spinal wide dynamic range neurons in response to peripheral stimuli, and melittin administration inhibited this neuronal activity. In behavioral assessment, analgesic effect of melittin was blocked by intrathecal α1- and α2- adrenergic receptor antagonists administration. Based on these results, we suggest that melittin could be used as an analgesic on oxaliplatin-induced peripheral neuropathy, and that its effect is mediated by activating the spinal α1- and α2-adrenergic receptors.

## 1. Introduction

Oxaliplatin is the third-generation platinum based chemotherapeutic agent, which is combined with fluorouracil and leucovorin for metastatic colorectal cancer [1]. Also, it has been proven to be effective for advanced esophagogastric [2] and pancreatic cancer [3]. However, oxaliplatin treatment can induce peripheral neuropathy expressed in sensitivity to cold, numbness and tingling in hands and feet [4,5]. These sensory neuropathies have long been recognized as the major dose-limiting adverse events of oxaliplatin treatment [6]. So far, several agents showed potentiality to prevent and treat oxaliplatin-induced peripheral neuropathy [7,8]. However, these agents also have limitations including side effects such as fatigue, insomnia, and nausea [8].

Melittin, which consists of 26 amino acids, is a major component of bee venom [9,10]. Bee venom has been shown to be effective on a variety of pain models, such as cancer pain [11], inflammatory pain [12], and neuropathic pain [13]. In our previous study, we demonstrated that subcutaneous bee venom (1 mg/kg) and melittin (0.5 mg/kg) administration on ipsilateral ST36 alleviated paclitaxel-induced mechanical allodynia [14]. Also, it was reported that melittin injection on ST36 reduced pain caused by complete Freund’s adjuvant-induced rheumatoid arthritis [15]. However, the analgesic effect of melittin on oxaliplatin-induced peripheral neuropathy has not been studied yet, and its neuropharmacological mechanism remains undiscovered.

In this study, first, we conducted behavioral tests to assess whether melittin can relieve oxaliplatin-induced mechanical and cold allodynia in rats. Secondly, by using in vivo electrophysiological method, we observed the activities of wide dynamic range (WDR) neurons in the spinal cord after oxaliplatin and melittin injection. Finally, by administrating receptor antagonists intrathecally, we determined whether spinal adrenergic receptors, which are known to be the key mechanisms of bee venom analgesia, are involved in the effect of melittin.

## 2. Results

### 2.1. Mechanical and Cold Allodynia Induced by Oxaliplatin Administration in Rats

**Figure 1:**
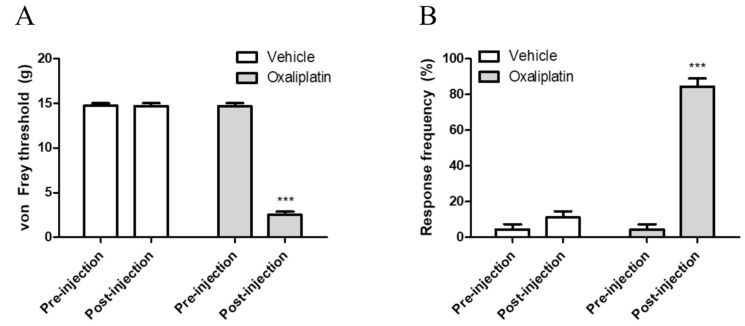
Oxaliplatin administration induces increased behavioral response to mechanical and cold stimulation. Three days after intraperitoneal injection of 6 mg/kg of oxaliplatin, mechanical (**A**) and cold (**B**) allodynia were induced. Behavioral tests were conducted by using von Frey filament and acetone, to assess mechanical and cold allodynia, respectively. Nine rats were allocated in each group. Data is presented as the mean ± standard error of the mean (S.E.M); *** *p* < 0.001, by Bonferroni post-test after two-way analysis of variance (ANOVA).

Single intraperitoneal injection of oxaliplatin at a dose of 6 mg/kg induced mechanical and cold allodynia in rats. As reported in our previous studies, these behavioral changes were shown significantly from three to seven days after the injection [16,17]. Figure 1A shows the results of behavior response to von Frey filament stimuli, exhibiting lowered withdrawal threshold after oxaliplatin administration. Responses to cold stimuli (10 μL of acetone application on ventral side of right hind paw) were also exaggerated in terms of intensity (Figure 1B). We interpreted these deteriorated responses to peripheral stimuli as mechanical and cold allodynia. 

### 2.2. Melittin Alleviates Oxaliplatin Induced Mechanical and Cold Allodynia

**Figure 2:**
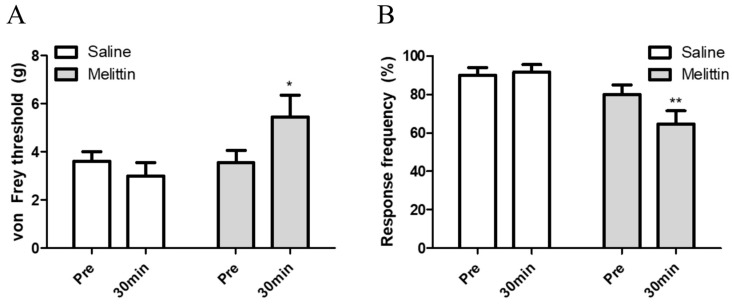
Subcutaneous melittin injection on acupoint ST36 alleviated mechanical and cold allodynia caused by oxaliplatin injection. Rats showing significant mechanical and cold allodynia after oxaliplatin injection were divided into two groups. Saline (*n* = 13) and melittin (*n* = 18). Melittin alleviated both the mechanical allodynia (**A**) and cold allodynia (**B**). Data is presented as the mean ± S.E.M.; * *p* < 0.05, ** *p* < 0.01; by Bonferroni post-test after two-way ANOVA.

To verify the analgesic effect of melittin on oxaliplatin-induced peripheral neuropathy, 0.5 mg/kg of melittin was injected subcutaneously on acupoint ST36. Behavioral assessments were done before and after melittin injection. Both the mechanical and cold allodynia were significantly attenuated 30 min after melittin injection (Figure 2A,B, respectively). These results indicate that subcutaneous melittin injection on ipsilateral acupoint can alleviates oxaliplatin-induced peripheral neuropathic pain.

### 2.3. Mellitin Inhibits Oxaliplatin-Induced Hyperexcitated Spinal WDR Neuronal Activity

**Figure 3:**
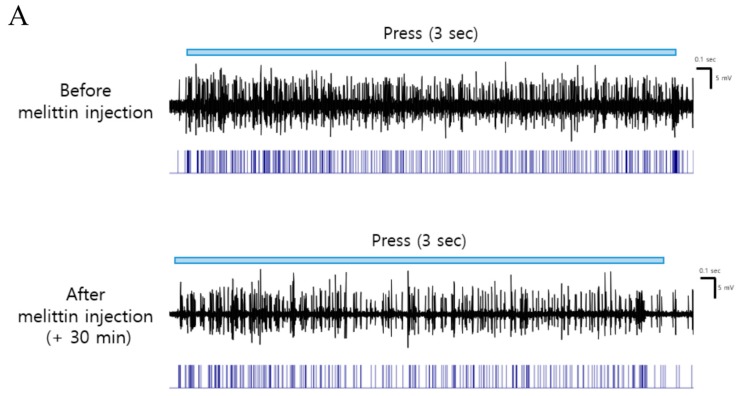

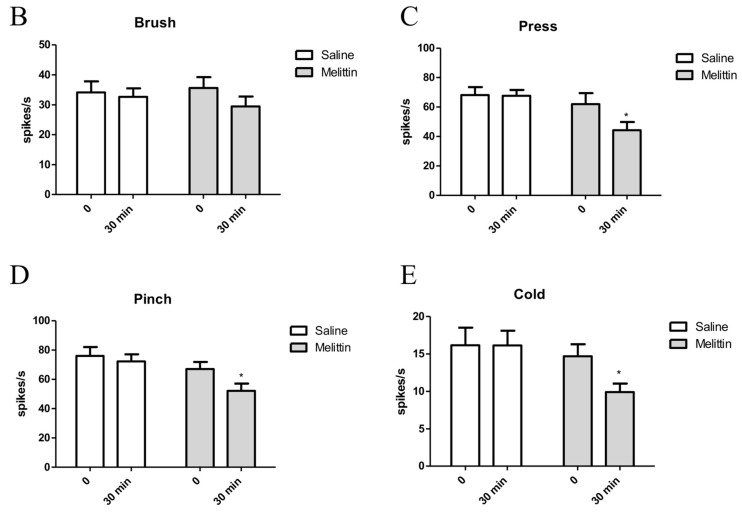
Inhibitory effects of melittin on increased firing of spinal WDR neurons in response to peripheral stimulation in oxaliplatin-injected rats. (**A**) Representative raw trace of spinal WDR neuronal activity altered by melittin injection. (**B–E**) Spike numbers of spinal WDR neuron reacting to peripheral stimuli (brush, press, pinch, and cold) 30 min after 0.5 mg/kg of melittin administration. *N* = 11 for each group. Data is presented as the mean ± SEM.; * *p* < 0.05; by Bonferroni post-test after two-way ANOVA (B–E).

We investigated whether melittin could inhibit the increased spinal WDR neuronal activity to peripheral stimuli after oxaliplatin injection. Figure 3A shows the representative raw trace of the spinal WDR neuronal responding to three seconds of pressure before and after melittin injection. Melittin significantly inhibited mechanical (press and pinch, but not brush) and cold (acetone drop) stimuli, which is quantified by spike number decrement (Figure 3B–E). These electrophysiological results correlate to melittin analgesia shown in behavioral assessment (Figure 2).

### 2.4. Both Spinal α1- and 2- Adrenergic Receptors are Involved in the Analgesic Effects of Melittin

**Figure 4:**
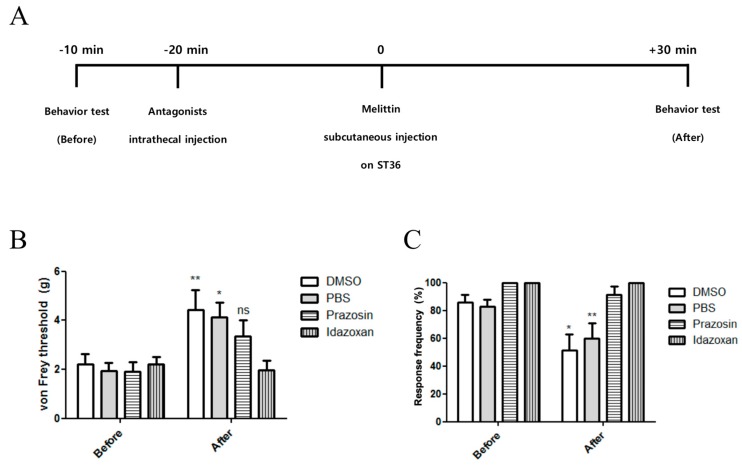
Intrathecal α-adrenergic receptor antagonists reversed the analgesic effect of melittin on mechanical and cold allodynia. (**A**) Timeline of behavioral test conducted with adrenergic antagonists injection. (**B**,**C**) Both α1 and α2-adrenergic receptor antagonists blocked the analgesic effect of melittin. All drugs were injected at ST36. DMSO and PBS were used as control to prazosin and idazoxan, respectively. *N* = 7 for each group. Behavioral tests were conducted 30 min after the melittin administration. Data are presented as mean ± SEM; ns; non-significant, * *p* < 0.05, ** *p* < 0.01; by Bonferroni post-test after two-way ANOVA.

To elucidate the spinal mechanism of the analgesic effect of melittin, α-adrenergic receptor antagonists were injected intrathecally before melittin injection. Figure 4A represents the time schedule of the behavioral study. Prazosin (α1-adrenergic receptor antagonist, 30 μg, i.t.) or idazoxan (α2-adrenergic receptor antagonist, 50 μg, i.t.) was administered 20 min before treatments. Both prazosin and idazoxan blocked the melittin analgesia on mechanical and cold allodynia (Figure 4B,C). In contrast, the control of these antagonists (Dimethyl sulfoxide (DMSO) and phosphate-buffered saline (PBS), respectively) did not cause any significant effect on the analgesic effect of melittin. Taken together, both spinal α1- and 2-adrenergic receptors are shown to be involved in the analgesia action of melittin on oxaliplatin-induced peripheral neuropathy.

## 3. Discussion

Oxaliplatin has a broad spectrum of anticancer activity and a better safety profile than cisplatin, which is the first platinum based drug to enter clinical use, but with a significant side toxicity [18]. Nonetheless, oxaliplatin-induced peripheral neuropathy, characterized by mechanical and cold allodynia, could be the main cause of dose reduction and treatment cessation [19]. Although a variety of agents including opioids, antidepressant, antiepileptics, or topical liniments are used to manage this neuropathy; so far, there is no ideal therapeutic agent due to their own side effects or low efficacy [20]. Thus, it would be valuable to discover novel analgesics with satisfactory efficacy and minimal side effects.

For several years, our lab has focused on the analgesic effects of bee venom acupuncture on various chemotherapy-induced peripheral neuropathic pain [14,16,21,22,23]. In one of our previous studies conducted on paclitaxel-induced peripheral neuropathy animal model, both bee venom acupuncture and melittin were shown to significantly attenuate the pain behavior [14]. It was clear that the mechanism of bee venom acupuncture analgesia involves the spinal α2-adrenergic receptor activation, however, the spinal mechanism of melittin analgesia remained unclear.

In this study, we showed that single oxaliplatin injection (6 mg/kg) produced mechanical and cold allodynia in rats (Figure 1). Subcutaneous melittin injection on ST36 alleviated pain response of ipsilateral hind paw to mechanical and cold stimuli (Figure 2). Like the results of behavioral assessments, on in vivo electrophysiological study, spike numbers of hyperexcitated spinal WDR neuron in response to peripheral mechanical and cold stimuli were inhibited by melittin administration (Figure 3). Furthermore, the analgesic effect of melittin was blocked by intrathecal adrenergic receptor antagonists, both α1 and α2 (Figure 4).

In the spinal dorsal horn, all α1, α2, and β adrenergic receptors are present. Especially, activation of α1 and α2-adrenergic receptor are known to be able to mediate the anti-nociceptive action: α1-adrenergic receptor has an excitatory effect on inhibitory interneurons, which can increase the release of inhibitory transmitters, while α2-adrenergic receptor has an inhibitory effect by decreasing the activation of both Aδ and C afferent fibers [24]. Based on our previous study [17], it was shown that oxaliplatin-induced allodynic behavior and spinal neuronal hyperexcitation can be modulated by spinal noradrenaline or its receptor agonists. Inhibitory efficacy was shown in α2 and α1-adrenergic receptor agonists, but not in β. Between α2 and α1, α2-adrenergic receptor agonists showed a greater inhibitory effect. This result implies that the activation of spinal noradrenergic inhibitory system can modulate the oxaliplatin-induced peripheral neuropathy. Although the mechanism of bee venom analgesia depends on the pain model employed, spinal α2-adrenergic receptor has been reported to be generally involved [14,25,26]. Phospholipase A2, another major component of bee venom, also induced analgesic effect in oxaliplatin-induced peripheral neuropathy and its action was blocked by intraperitoneal α2-, but not by α1-adrenergic receptor antagonist [27].

In this study, we discovered the potentiality of melittin as an analgesic agent in chemotherapy induced peripheral neuropathy. So far, melittin has been regarded as a pain producing substance, because of its strong surface activity on lipid membranes followed by releasing inflammatory mediators and activating primary nociceptor cells [28]. Actually, subcutaneous injection of melittin on posterior surface of hind paw produced spontaneous paw flinching reflex and an increase in the frequency of the spinal WDR neuron’s spontaneous discharges on cutaneous receptive field of hind paw [29]. However, in our behavior study (Figure 2), subcutaneous melittin administration on ST36 did not induce spontaneous painful behavior. Furthermore, it did not generate spontaneous discharge of WDR neuron responding to receptive filed of hind paw. ST36 is the representative acupoint of Korean medicine, which has been reported to be effective on relieving various pain [30,31]. As other papers reported that the analgesic effect of acupuncture or electroacupuncture on ST36 was mediated by activating the descending pain inhibitory system [32,33,34], melittin injection on ST36 may also have activated the noradrenergic descending inhibitory system.

The advantage of injecting melittin on ipsilateral acupoint than intraperitoneal has two strong points. First, ST36 is anatomically located near the ascending nerve pathways from hind limb, thus the analgesic effect could be more efficient than systemic administration. The other is minimizing adverse effect of melittin. Systemic injection such as intravenous or intraperitoneal might be accompanied by hemolysis. Although the cytotoxicity of melittin is dependent on its concentration, given that it has affinity to erythrocyte membrane, intradermal or subcutaneous injection of low dose of melittin may be a safe method to prevent hemolysis [35].

Although there is no clinical trial that assessed the effect of melittin on humans, Park et al. investigated the effect and safety of sweet bee venom pharmacoacupuncture on five patients with chemotherapy-induced peripheral neuropathy (CIPN) [36]. Patients’ visual analogue scale and world health organization (WHO) CIPN grade as primary results were shown to be effective without causing significant adverse effects such as allergic reaction. As the major component of sweet bee venom is melittin, this result shows that melittin may be used safely to patient in the future

Furthermore, another critical value of melittin as an analgesic dealing with chemotherapy-induced peripheral neuropathy is that it has an anticancer activity [9]. Melittin exerted its anticancer effect by modulating tumor-associated macrophage, which inhibited tumor angiogenesis without non selective cytotoxicity when injected at low dose (0.5 mg/kg) [37]. Therefore, as the same dose of melittin significantly suppressed oxaliplatin-induced peripheral neuropathy in our experiments, we suggest that melittin could act as an adjuvant anticancer and an analgesic agent.

## 4. Conclusion

The results of the present study demonstrate that melittin administration on ST36 can relive the mechanical and cold allodynia induced by a single injection of oxaliplatin in rats. Furthermore, by in vivo electrophysiolgical study, we demonstrated that melittin can inhibit the hyperexcitation of spinal WDR neuron in response to peripheral stimuli. This analgesic effect of melittin was shown to be mediated by spinal α1 and α2-adrenergic receptor activation. Collectively, these results suggest that melittin has analgesic effect on oxaliplatin-induced peripheral neuropathy and the effect is mediated by activating the spinal α1 and α2-adrenergic receptor. Thus, based on these results, melittin could be used as an analgesic on oxaliplatin-induced peripheral neuropathy.

## 5. Materials and Methods

### 5.1. Animals

Sprague-Dawley (SD) rats (7–8 weeks old, 180–210 g, *n*= 99 in total) were purchased from Young Bio (Gyeonggi, Korea) and housed in cages with free water and food. The room was maintained with a 12 h light/dark cycle and kept at 23 ± 2 °C. All procedures involving animals were approved by the Institutional Animal Care and Use Committee of Kyung Hee University (KHUASP(SE)-19-047; approved 12 June 2019, KHUASP(SE)-18-153; approved 29 January 2019) and performed according to the ethical guidelines of the International Association Of the Study of Pain [38]. At the end of the study, the animals were killed by injecting an overdose of urethane.

### 5.2. Oxaliplatin Administration

Oxaliplatin (Wako Pure Chemical Industries, Osaka, Japan) was dissolved in a 5% glucose solution at a concentration of 2 mg/mL and was intraperitoneally injected at a dose of 6 mg/kg [23]. The same volume of 5% glucose solution was injected to control group.

### 5.3. Behavioral Tests

Rats were habituated to the circumstances for 30 min before all behavioral tests. All experimenters conducting behavioral tests were blinded to the groups. Rats were placed on a wired mash and enclosed in a clear plastic box (20 × 20 × 14 cm).

To assess mechanical allodynia, paw withdrawal thresholds were measured by applying the von Frey filaments in the center of the right hind paw. Dixon’s up-down method and Chaplan’s calculation methods were used and withdrawal cut-off values was 15 g [39,40].

Cold allodynia test was conducted as in our previous study [17]. Acetone (10 μL) was applied to the ventral surface of the right hind paw five times by using a pipette with polyethylene tube, and the experimenter monitored the behavioral response for 40 s [41,42].

### 5.4. Melittin Administration

Melittin (Melittin from honey bee venom; Sigma, St. Louis, MO, USA; 0.5 mg/kg) was dissolved in saline [14], and it was injected once subcutaneously on ipsilateral acupoint, Zusanli (ST 36) of right leg between three and seven days after oxaliplatin administration in rats showing allodynic behavior. ST36 is located in the anterior tibial muscle, 5 mm lateral and distal to the anterior tubercle of the tibia [43].

### 5.5. In vivo Extracellular Recording

In vivo extracellular recording was done according to our previous studies [17,44,45]. Briefly, rats were anesthetized with urethane (Sigma, St. Louis, MO, USA; 1.2–1.5 g/kg, intraperitoneal (i.p.)) and the procedures were conducted on a warm plate for maintenance of body temperature. Thoracolumbar vertebral laminectomy was performed at the level of T13–L2 to expose the spinal segment L3–L5. Once the laminectomy was done, the rats were placed in a stereotaxic apparatus to fix the vertebrae. On the surface of exposed spinal cord, Krebs solution (in mM: 117 NaCl, 3.6 KCl, 2.5 CaCl_2_, 1.2 MgCl_2_, 1.2 NaH_2_PO_4_, 11 glucose and 25 NaHCO_3_) with oxygenated of 95% O_2_-5% CO_2_ gas was continuously irrigated at a flow rate of 10 to 15 mL/min at 38 ± 1 °C. With the solution perfused, dura mater was removed, and pia-arachnoid mater was cut ot make a small opening to insert the tungsten electrode (impedance of 10 MΩ, FHC, ME, USA) smoothly without adjacent tissue suppression.

To identify the receptive filed of WDR neurons, electrode was inserted slowly into the dorsal horn while stimulating the hind paw with light touch (brushing or tapping), pinching (forceps), and acetone drop. After determining the receptive field, brush stimulus was given by applying the camel brush 5 times during 3 s. Press stimulus was given by pressing the center of the receptive field for 3 s, using the blunt tip of the brush with a diameter of 0.5 cm and a magnitude of about 20 g. Pinch stimulus was done by pinching the skin with toothed forceps (11022-14, Fine Science Tools, Heidelberg, Germany) for 3 s. For cold stimulation, 10 μL of acetone drop was applied to the receptive field. Recorded action potentials were amplified with the bioamplifier (DAM80, WPI, Sarasota, FL, USA). The data were digitized (Digidata 1440A, Axon instruments, Foster City, CA, USA) and stored in a personal computer using pClamp 10 software (Axon instruments, Foster City, CA, USA). Recorded data were spiked-sorted with Spike2 (version 6, Cambridge Electronic Design, Cambridge, UK) and produced spike number.

### 5.6. Antagonist Treatment

To investigate the spinal involvement of noradrenergic receptors, oxaliplatin administered rats were divided randomly into four groups: dimethyl sulfoxide (DMSO; Sigma, St. Louis, MO, USA) + melittin, prazosin + melittin, PBS + melittin, and idazoxan + melittin. α1-adrenoceptor antagonist prazosin (Sigma, St. Louis, MO, USA; 30 μg) was dissolved in 20% DMSO. α2-adrenoceptor antagonist idazoxan (Sigma, St. Louis, MO, USA; 50 μg) was dissolved in PBS. Under isoflurane anesthesia (Hana Pharm. Co., Hwaseong-si, Kyeonggi-Do, Korea), all antagonists were treated intrathecally with a direct lumbar puncture as previously described [14,16,17]. 

### 5.7. Statistics

Statistical analysis was conducted with the software of Prism 5.0 (GraphPad software, La Jolla, CA, USA, 2008). All data are presented as the mean ± SEM. *p* < 0.05 was considered significant.
